# Continuous wavelet transform for solving the problem of minor components in quantitation of pharmaceuticals: a case study on the mixture of ibuprofen and phenylephrine with its degradation products

**DOI:** 10.1186/s13065-023-01059-1

**Published:** 2023-10-24

**Authors:** Said A. Hassan, Reham A. Fekry, Yasmin M. Fayez, Khadiga M. Kelani

**Affiliations:** 1 Analytical Chemistry Department, Faculty of Pharmacy, Cairo University, Kasr El-Aini Street, Cairo, 11562 Egypt; 2 Analytical Chemistry Department, Faculty of Pharmacy, Modern University for Technology and Information, El-Hadaba El-Wosta, Mokatam, 5th district, Cairo, Egypt

**Keywords:** Derivative ratio-zero crossing, Derivative spectrophotometry, Ibuprofen, Minor component, Numerical differentiation, Phenylephrine, Wavelet transform

## Abstract

The presence of minor components represents a challenging problem in spectrophotometric analysis of pharmaceuticals. If one component has a low absorptivity or present in a low concentration compared to the other components, this will hinder its quantitation by spectrophotometric methods. Continuous Wavelet Transform (CWT) as a signal processing technique was utilized to figure out a solution to such a problem. A comparative study was established between traditional derivative spectrophotometry (Numerical Differentiation, ND) and CWT to indicate the advantages and limitations of each technique and possibility of solving the problem of minor components. A mixture of ibuprofen (IBU) and phenylephrine (PHE) with its degradation products forming a ternary mixture was used for comparing the two techniques. The two techniques were applied on raw spectral data and on ratio spectra data resulting in four methods, namely ND, CWT, Derivative Ratio-Zero Crossing (DRZC) and Continuous Wavelet Transform Ratio-Zero Crossing (CWTR-ZC) methods. By comparing the results in laboratory prepared mixtures, CWT technique showed advantages in analysis of mixtures with minor components than ND. The proposed methods were validated according to the ICH guideline Q2(R1), where their linearity was established with correlation coefficient ranging from 0.9995 to 0.9999. The linearity was in the range 3–40 μg/mL for PHE in all methods, while for IBU it was 20–180 and 30–180 μg/mL in CWT and ND methods, respectively. The CWT methods were applied for quantitative determination of the drugs in their dosage form showing the ability of the methods to quantitate minor components in pharmaceutical formulations.

## Introduction

Spectrophotometry is one of the most commonly used techniques due to the availability of its instruments, simplicity and speed. UV–vis absorption spectroscopy is a well-established technique for rapid and accurate determination of analytes in mixtures without prior separation if the interferences between the spectra can be eliminated.

Several techniques can be used for elimination of interferences in spectroscopy such as mathematical manipulations [1–3], chemometrics [4–6] and signal processing. The most commonly used techniques are signal processing ones, of which the most popular is Derivative Spectrophotometry (DS). The first use of DS was in mass spectroscopy [7], then it was applied to UV–vis and IR spectroscopy [8]. DS is a powerful method used in analytical chemistry to eliminate background interference, resolve spectra, sharpen peaks and carry out quantitative analysis. It has a significant role in resolution of overlapped UV–vis spectra [9]. In spectral analysis, application of ND to the absorption spectra has several drawbacks such as: diminishing peak intensity, requiring smooth and scaling factor functions. Therefore, the obtained derivative spectra are deformed from the original ones, and consequently, the traditional ND may generate errors in analysis [10].

As a result, alternatives for derivative calculation were suggested to overcome these drawbacks. Least-squares procedures for the smoothing and differentiation of spectral data were introduced by Savitzky and Golay [11]. Wahbi et al. [12] applied Fourier functions for spectrophotometric quantitation of mixtures (discrete Fourier transform). Mean centering was also introduced as a processing technique for ratio spectra, and it was applied for the analysis of binary and ternary mixtures [13–15]. Finally, wavelet transform represents a powerful tool for signal processing that can be used for spectrophotometric analysis of mixtures [16].

The wavelet transform process performs decomposition of the spectrum into simpler, fixed building blocks at different scales and positions [16]. In the field of UV–vis spectrophotometry, Continuous wavelet transform (CWT) combined either with a zero-crossing technique or ratio spectra was used for simultaneous determination of chemical species in binary and ternary mixtures [17–19].

In addition to the common problem of spectral overlapping and interference that hinders the use of spectrophotometric methods in the analysis of pharmaceutical mixtures, the problem of minor components arises as a new challenge for these methods of analysis. This problem arises when the pharmaceutical formulation contains one drug in a low concentration or has very low absorptivity compared to the other components. This will make the resolution of the spectrum of such drug more difficult and will require special treatment of the dosage form during analysis to allow its quantitation such as spiking technique [20, 21]. The need for higher amounts of standards and the tedious process are the reasons why spiking is no longer the ideal solution for such a problem. New approaches emerged for solving this challenging problem such as absorptivity target concentration values [22], response correlation and advanced balance point-spectrum subtraction methods [23].

**Fig. 1:**
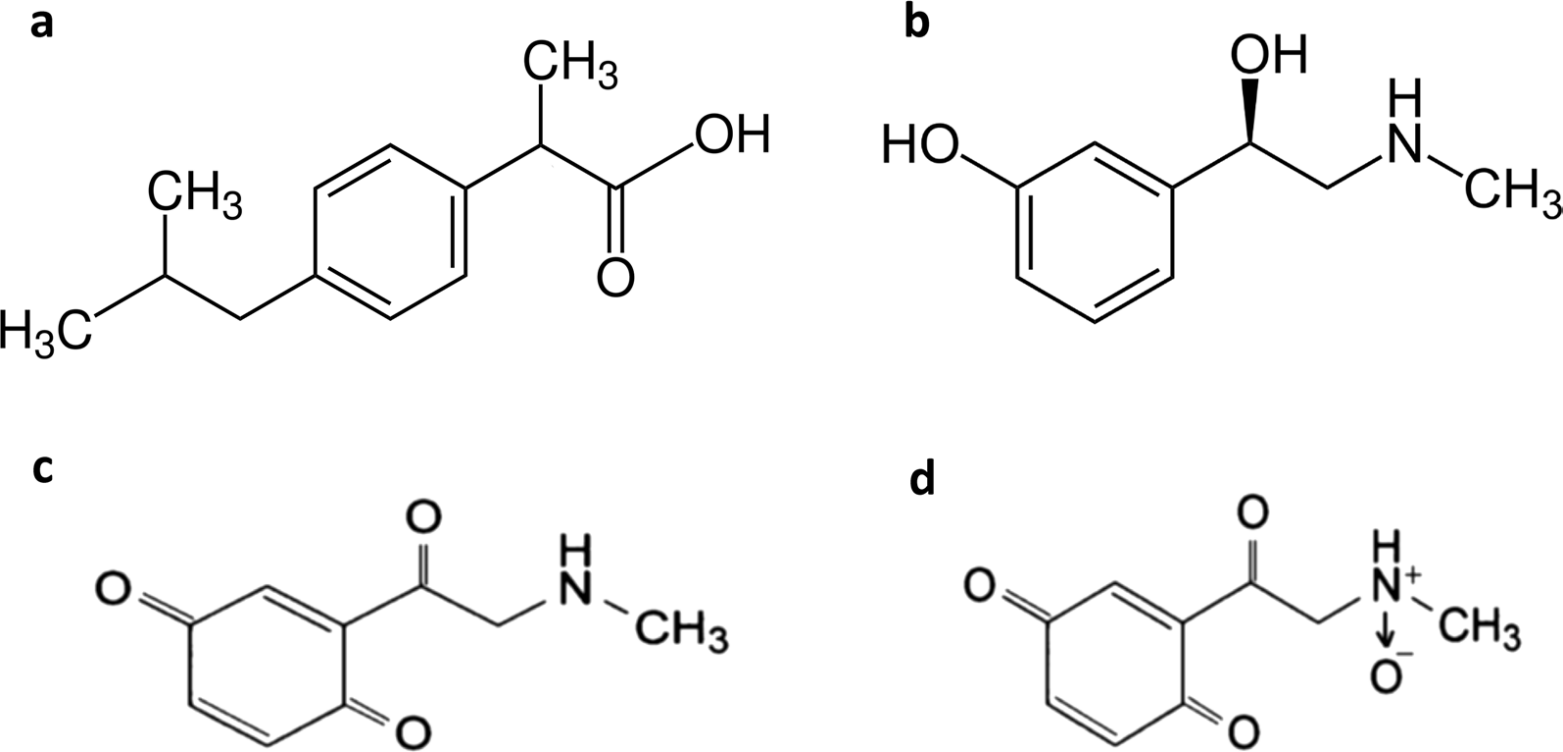
Chemical structures of **a** IBU, **b** PHE **c** PHEdeg1, and **d** PHEdeg2

Ibuprofen (IBU), (2RS)-2-[4-(2-Methylpropyl)phenyl]propanoic acid [24] (Fig. 1), is a nonsteroidal anti-inflammatory drug that is used for treating fever and pain. It acts mainly through inhibiting the cyclooxygenase-2 enzyme [25]. Phenylephrine (PHE), (1R)-1-(3-Hydroxyphenyl)-2-(methylamino)ethanol [24] (Fig. 1), is primarily used as a decongestant, to dilate the pupil, to increase blood pressure, and to relieve hemorrhoids [26]. The combination dosage form of IBU and PHE is used to treat stuffy nose, sinus congestion, headache, fever, and minor aches and pains caused by the common cold and flu [27]. It is available in the market in the form of coated tablets under trade name Grippostad^®^. Each tablet of Grippostad^®^ contains 200 mg of IBU and 5 mg of PHE, the ratio is 40:1. This ratio is a prime example of the problem of minor components.

Scientific reports showed several degradation studies using multi-component UV–vis spectroscopy and estimation of NSAIDs [28–31]. Yet, few methods were reported for assaying IBU and PHE simultaneously in their binary combination [32–35]. The two drugs were subjected to stability studies in their mixtures with other drugs [36–42], but no method was developed for the stability indicating assay of this binary mixture. During literature survey, IBU was found to be susceptible to strong oxidative and thermal degradation [40–42]. PHE was reported to be more stable toward acidic, alkaline, and thermal conditions [36–39]. PHE is also more susceptible to oxidative conditions producing 2 degradation products [37]. PHE photodegradation may produce epinephrine [43], which has stronger agonist effect on adrenergic receptors [44]. This fact indicates that possible degradation of PHE may cause severe side effects to patients. Degradation of PHE may also cause physical instability as it may be accompanied by a change in color even in light-protected media [45]. Acetylated degradation products of PHE in tablets containing aspirin were detected and characterized [46]. Phenolic cyclization is a possible decomposition pathway for PHE to form tetrahydroisoquinolines [47], which are known to induce Parkinson’s disease [48].

From these facts, it’s proven that degradation of PHE is a major health concern in pharmaceutical formulation’s development in comparison to IBU. Therefore, PHE degradation is the focus in our study.

In this work, ND and CWT techniques were applied on raw data (ND and CWT methods) and on ratio spectra data (Derivative Ratio-Zero Crossing “DRZC” and Continuous Wavelet Transform-Zero Crossing “CWTR-ZC” methods) and the methods were compared regarding their ability to solve the problem of minor components in spectrophotometric analysis of pharmaceuticals. A mixture of IBU and PHE with PHE degradation products (PHEdeg) was used to present the comparison.

## Experimental

### Chemicals and materials

Methanol of HPLC grade was obtained from Sigma Aldrich (Darmstadt, Germany). IBU was kindly supplied by Abbott laboratories (Cairo, Egypt). PHE was kindly supplied by Amman Pharmaceutical Industries (Amman, Jordan). Their purities were found to be 99.62 ± 1.11% and 100.02 ± 1.14% for IBU and PHE, respectively, according to the BP methods [24]. Grippostad^®^ film coated tablets manufactured by Laboratorio Stada (Barcelona, Spain). Each tablet contains 200 mg of ibuprofen and 5 mg of phenylephrine.

### Instrumentation

Shimadzu dual beam UV–Vis spectrophotometer (Kyoto, Japan), UV-1650 PC model with bundle software. Processing of absorption and derivative spectra was done using version 3.7 of the UV PC personal spectroscopy program (Shimadzu, Kyoto, Japan). Scans have been performed at intervals between 200.0 nm to 400.0 nm at 0.2 nm with 1.00 cm quartz cells.

The CWT was performed using MATLAB^®^ 9.2.0.538062 (R2017a).

### Solutions

#### Preparation of PHE degradation product stock solution

Oxidative degradation was performed by refluxing 100.0 mg of PHE powder with 25 mL of 3% H_2_O_2_ for 3 h and protected from light and then excess H_2_O_2_ was removed by evaporation. Complete degradation was confirmed through TLC, the solution was cooled, transferred quantitatively into a 25-mL volumetric flask, and completed to volume using methanol to prepare stock solution of PHEdeg equivalent to 4 mg/mL PHE. Silica gel 60 F_254_ plates were used to check the disappearance of intact drug spot at R_f_ 0.12 and appearance of two spots of degradation products at R_f_ 0.45 and 0.55 using ethyl acetate–methanol-30% ammonia solution (8:2:0.1, by volume) as the developing mobile phase.

#### Solutions of IBU, PHE and PHEdeg

Standard stock solutions of 1.0 mg/mL of IBU and PHE were prepared from their standard powder using methanol. Stock solution of PHEdeg equivalent to 1 mg/mL PHE was prepared by dilution from previous degradation solution. Working solutions of 100 μg/mL of IBU, PHE and PHEdeg were prepared by suitable dilutions from the corresponding stock solutions.

### Procedures

#### Spectral characteristics of ibuprofen, phenylephrine and PHEdeg

The zero-order spectra of 120 μg/mL IBU, 3 μg/mL PHE and PHEdeg equivalent to 10 µg/mL PHE were scanned using methanol as a blank in the range 200–400 nm.

#### Calibration curves construction

Definite volumes of IBU and PHE working standard solutions (100 µg/mL) equivalent to 200–1800 µg and 30–400 µg, respectively, were accurately transferred into two separate series of 10-mL volumetric flasks and methanol was used to complete the volume of each flask. The absorption spectra were scanned using methanol as blank.

##### Numerical differentiation (ND)

For IBU, fourth derivative spectra were calculated using Δλ = 8 nm and scaling factor = 1000. For PHE, second derivative spectra were calculated using Δλ = 8 nm and scaling factor = 1000. Calibration curves were constructed relating the peak amplitude of the corresponding derivative spectra at 272.4 and 275.4 nm to the corresponding concentrations of IBU and PHE, respectively.

##### Continuous wavelet transform (CWT)

For IBU, CWT spectra were calculated using Morlet wavelet family (morl) with scale = 30, while for PHE Gaussian-5 family (gaus-5) with scale = 100 was used. The amplitudes at 264.6 and 288.0 nm were plotted against the corresponding concentrations of IBU and PHE, respectively.

##### First derivative of ratio spectra (DD1)

The spectrum of PHEdeg equivalent to 30 μg/mL PHE was used as divisor for PHE. The first derivative of these ratio spectra was calculated with Δλ = 4 nm and scaling factor = 10. Calibration curve was constructed relating the peak amplitude at 270.8 nm to the corresponding concentrations of PHE.

##### Continuous wavelet transform ratio-zero crossing (CWTR-ZC)

For IBU, the spectrum of 40 μg/mL PHE was used as divisor, then the CWT of these spectra were calculated using morl family with scale = 20. For PHE, the spectrum of PHEdeg equivalent to 30 μg/mL PHE was used as divisor, and the CWT of these spectra were calculated using morl family with scale = 50. Calibration curves were constructed relating the peak amplitude of the corresponding spectra at 263.8 and 282.6 nm to the corresponding concentrations of IBU and PHE, respectively.

#### Application of the signal processing methods for the quantitation of IBU and PHE in laboratory-prepared mixtures

Different volumes of IBU, PHE and PHEdeg were taken from their working solutions into a single series of 10-mL measuring flasks, the formed mixtures contained diverse ratios of the two drugs and different percent of degradation products. Concentrations of IBU and PHE were calculated using the previous procedures.

#### Application of the proposed methods for the determination of IBU and PHE in Grippostad^®^ tablets

Ten of Grippostad^®^ film coated tablets (labelled to contain 200 mg of IBU and 5 mg PHE per tablet) were precisely weighed, the tablets were first stripped of the film before being finely powdered. A precisely weighed portion containing 200 mg of IBU and 5 mg of PHE was sonicated in 30 mL of methanol for 10 min before being filtered into a 100-mL volumetric flask. The residues were rinsed multiple times each with 10 mL of methanol, and the solution was adjusted to the mark by the same solvent. An aliquot was then diluted in order to prepare a solution containing 120 and 3 µg/mL of IBU and PHE, respectively.

## Results and discussion

### The problem of minor component in the studied mixture

**Fig. 2:**
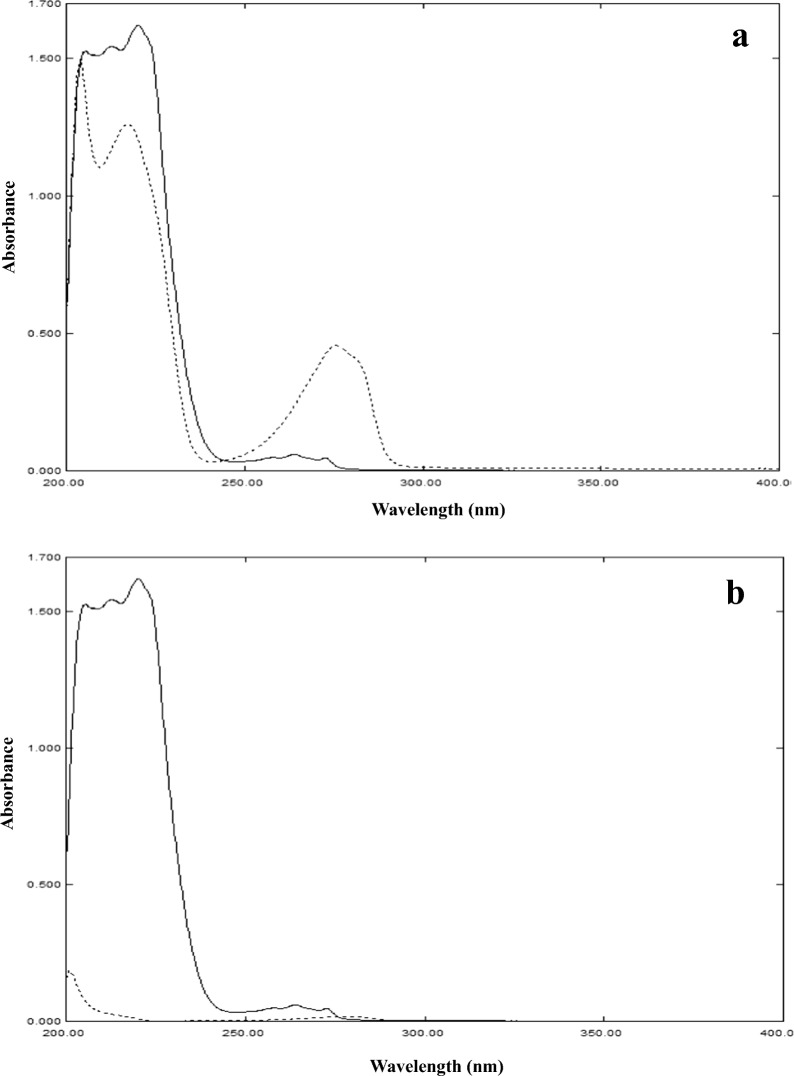
Zero order absorption spectra of **a** 40 µg/mL IBU (─) and 40 µg/mL PHE (....) and **b** 40 µg/mL IBU (─) and 1 µg/mL PHE (....) using methanol as blank

The objective of this work was to establish a comparative study between two signal processing techniques and show the advantages and weaknesses of these techniques in analysis of mixtures with minor components. A mixture of IBU and PHE was chosen for demonstration of this comparison, the two drugs are combined in Grippostad® tablets for the relief of cold symptoms. Each tablet contains 200 mg of IBU and 5 mg of PHE, this large difference in concentration (40:1) with the similar absorptivity of the two drugs as shown in Fig. 2a represent a challenge for the spectrophotometric analysis of these tablets in QC laboratories.

**Fig. 3:**
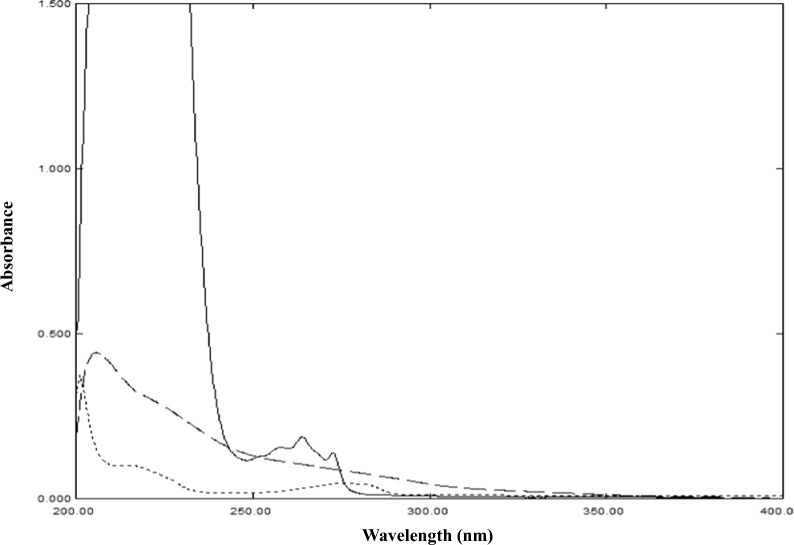
Zero order absorption spectra of 120 µg/mL IBU (─), 3 µg/mL PHE (....) and PHEdeg (----) equivalent to 10 µg/mL PHE using methanol as blank

The main challenge is to quantify PHE in presence of the large amount and hence large absorbance of IBU as shown in Fig. 2b. PHE concentration in Fig. 2b spectrum is 1 µg/mL and shows absorbance below the accepted limit at its two λ_max_ (217.0 and 273.0 nm). To overcome the problem of non-linearity of PHE, the first concentration of PHE that can be used is 3 µg/mL, which means in dosage form solution, IBU concentration will be 120 µg/mL. At these concentrations, IBU will be out of linearity at its λ_max_ (220.0 nm), but at the second λ_max_ of 264.0 nm it will be within the accepted limit of absorbance. So, to solve this problem, the dosage form will be diluted to the concentration of IBU 120 µg/mL and PHE 3 µg/mL as shown in Fig. 3.

The methods used for analyzing this mixture are ND, CWT, DRZC and CWTR-RS, all these methods depend on the presence of a zero-crossing point. So, although the concentration ratio used (120:3) will solve the problem of spiking, a new challenge will arise as the wavelength range used will be limited to the range 240–300 nm instead of 200–300 nm. This means that during the development of the four methods, searching for the zero-crossing point will be limited to 60 nm instead to 100 nm representing a new challenge. This wavelength range (240–300 nm) already shows limited absorbance features, which increase the difficulty of developing the methods.

To simulate real situations in QC laboratories the PHE oxidative degradation products (PHEdeg) were added to the mixture. PHE is proved to produce two degradation products upon exposure to stress oxidative condition [37]. PHE was degraded and a stock solution of the two degradation products (Fig. 1) was prepared, whose concentration is expressed relative to PHE concentration used for its preparation. The PHEdeg spectrum represent the two degradation products found in degradation solution of PHE, it displays severe overlap with the spectra of IBU and PHE as shown in Fig. 3.

### Derivative methods (ND and CWT)

**Fig. 4:**
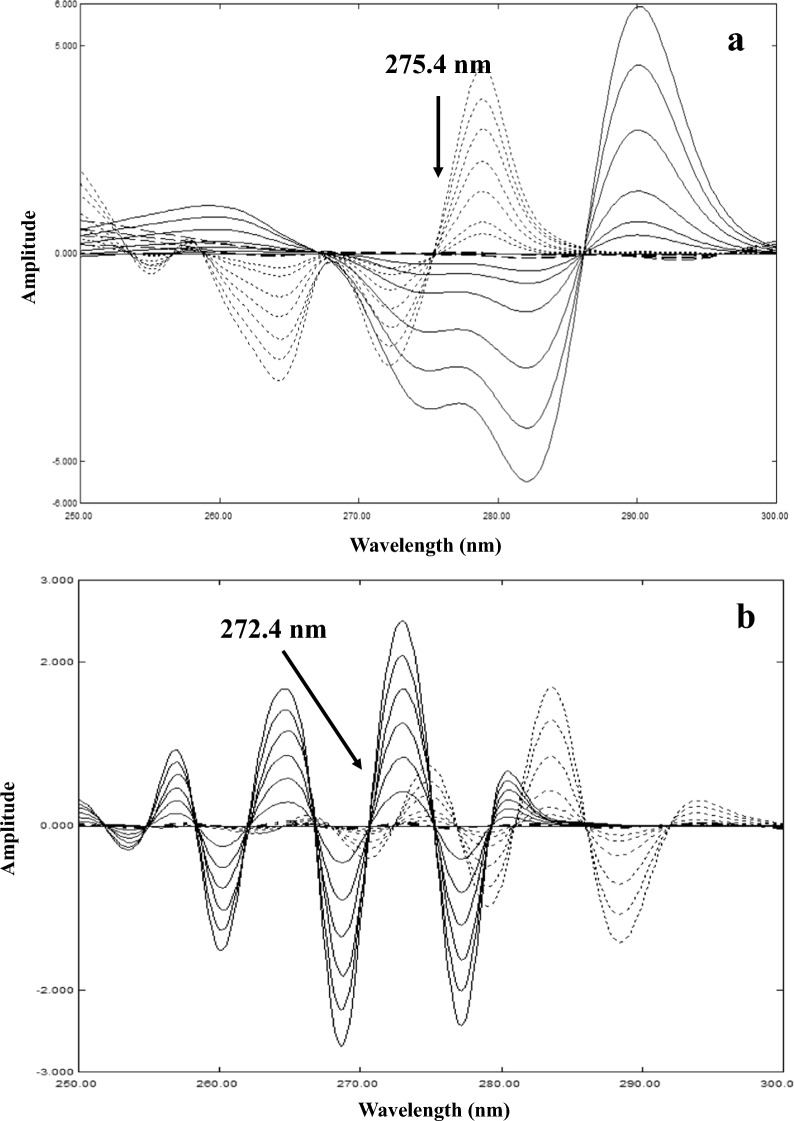
**a** Second derivative (ND) of 3–40 µg/mL PHE (──), 20–180 µg/mL IBU (....) and PHEdeg (----) equivalent to 10–40 µg/mL PHE showing zero-crossing point for PHE determination. **b** Fourth derivative (ND) of 30–180 µg/mL IBU (──), 3–40 µg/mL PHE (....) and PHEdeg (----) equivalent to 10–40 µg/mL PHE showing zero-crossing point for IBU determination

In ND method, no zero-crossing points were obtained in first order derivative for the two drugs. The second derivative was therefore calculated, where zero-crossing points were observed for determination of PHE as shown in Fig. 4a. The one that showed good linearity and was successfully used for its quantitation in laboratory prepared mixtures was 275.4 nm. In searching for a zero-crossing point for determination of IBU, third derivative was calculated and again no suitable zero-crossing points were observed. By calculating fourth order derivative spectra, zero-crossing points were obtained for IBU determination as shown in Fig. 4b. The best one regarding linearity and selectivity in laboratory prepared mixtures was 272.4 nm. For optimization of the derivative spectra, different Δλ (4, 8 and 16) with scaling factors of 10, 100 and 1000 were applied. The best parameters, regarding spectral shapes, linearity, and recovery, were Δλ = 8 and scaling factor of 1000.

**Fig. 5:**
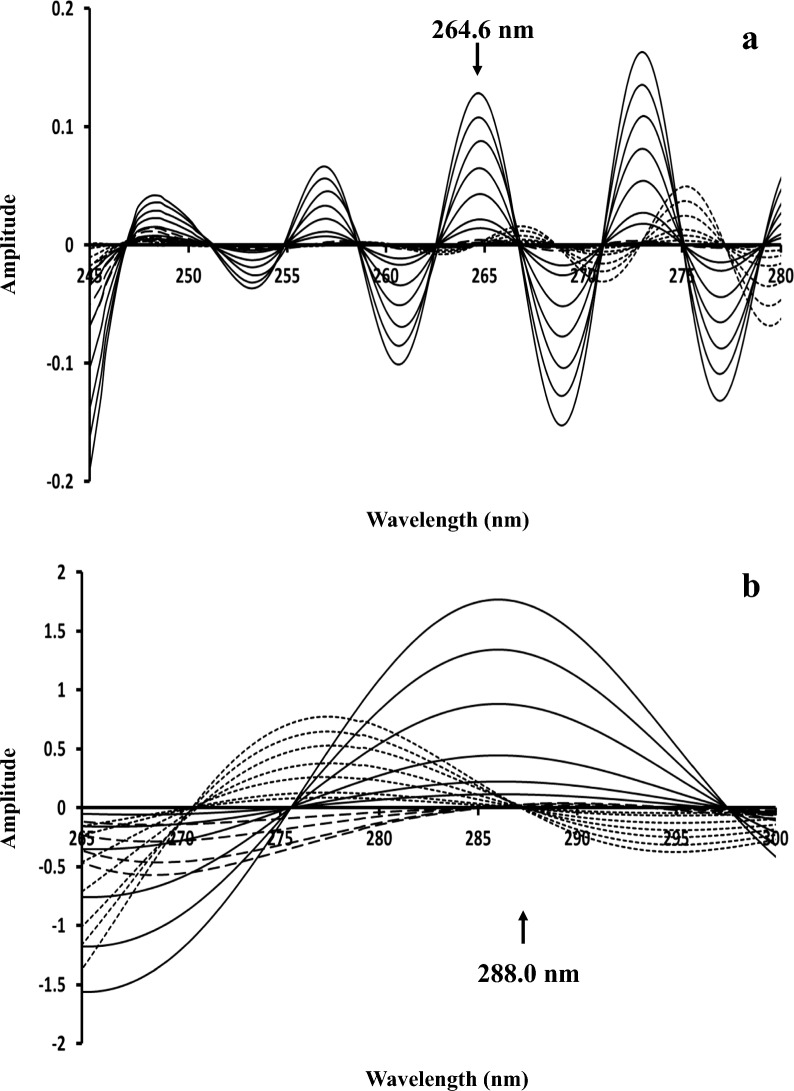
**a** Continuous Wavelet Transform (morl) of 20–180 µg/mL IBU (──), 3–40 µg/mL PHE (....) and PHEdeg (----) equivalent to 10–40 µg/mL PHE showing zero-crossing point for IBU determination. **b** Continuous Wavelet Transform (gaus-5) of 3–40 µg/mL PHE (──), 20–180 µg/mL IBU (....) and PHEdeg (----) equivalent to 10–40 µg/mL PHE showing zero-crossing point for PHE determination

In CWT method, for obtaining zero-crossing for the two drugs, different wavelet families were applied such as Daubechies (db), Morlet (morl), Coiflets (coif), Mexican hat (mexh), Meyer (meyr), Symlets (sym), and Gaussian (gaus). The families were tested in their different orders and with changing scale parameter. Zero-crossing points with the best linearity and recovery in laboratory prepared mixtures were obtained at 264.6 and 288.0 nm with family morl and gaus-5 for IBU and PHE, respectively (Fig. 5). The scale parameter was optimized for both families and morl family was best scaled at 30, while gaus-5 family was scaled at 100.

### Derivative ratio methods (DRZC and CWTR-ZC)

For DRZC and CWTR-ZC methods, the choice of optimum divisor is a main factor to optimize the method. For determination of IBU, different divisor spectra of PHE and PHEdeg were tested and the best one regarding shape, zero-crossing points, linearity, and recoveries in laboratory prepared mixtures was the spectrum of 40 µg/mL PHE (IBU/PHE 40). While for determination of PHE, different divisor spectra of IBU and PHEdeg were tested and the best one was the spectrum of PHEdeg equivalent to 30 µg/mL PHE (PHE/PHEdeg 30).

**Fig. 6:**
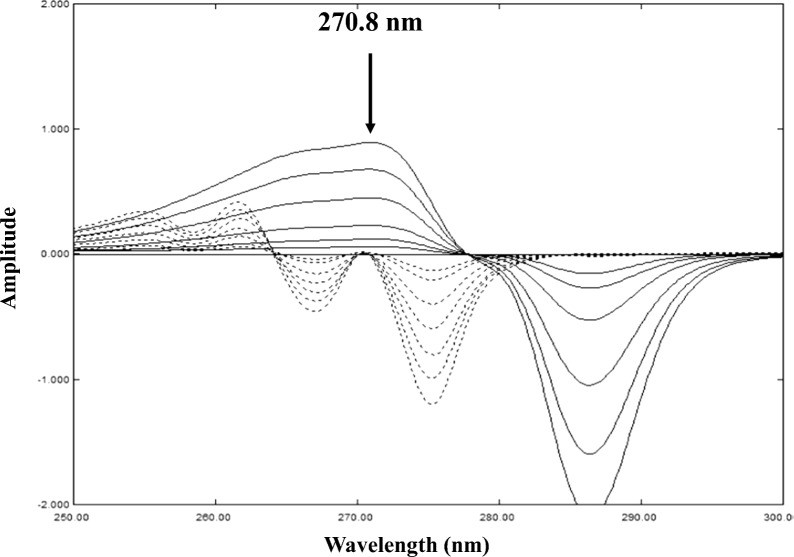
First derivative (ND) of ratio spectra (using PHEdeg equivalent to 30 µg/mL PHE as a divisor) of 3–40 µg/mL PHE (──) and 20–180 µg/mL IBU (....) showing zero-crossing point for PHE determination

For DRZC, after obtaining the best ratio spectra, first order derivatives for IBU and PHE were calculated using Δλ = 4 with a scaling factor of 10. The first derivative of ratio spectra (IBU/PHE 40) showed no possible zero-crossing points for IBU determination, the points observed showed bad recoveries in almost all the laboratory prepared mixtures. For PHE determination, zero-crossing points were observed in the first order derivative of ratio spectra (PHE/PHEdeg 30). The best wavelength regarding linearity and recovery was 270.8 nm (Fig. 6).

**Fig. 7:**
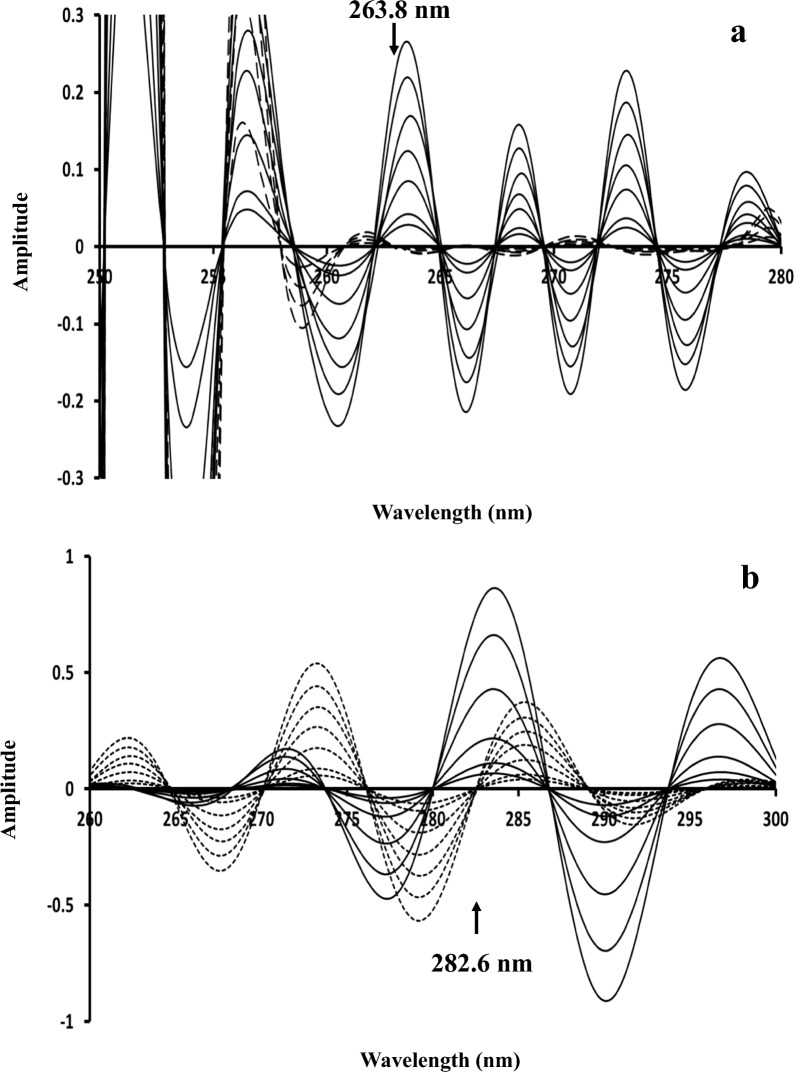
**a** Continuous Wavelet Transform (morl) of the ratio spectra (using 40 µg/mL PHE as divisor) of 20–180 µg/mL IBU (──) and PHEdeg (----) equivalent to 10–40 µg/ml PHE showing zero-crossing point for IBU determination. **b** Continuous Wavelet Transform (morl) of the ratio spectra (using PHEdeg equivalent to 30 µg/mL PHE as a divisor) of 3–40 µg/mL PHE (──) and 20–180 µg/mL IBU (....) showing zero-crossing point for PHE determination

For CWTR-ZC, several wavelet families and scales were applied on the ratio spectra IBU/PHE 40 and PHE/PHEdeg 30. For IBU determination, morl family with scale parameter of 20 was used on IBU/PHE 40 ratio spectra, and several zero-crossing points were observed and the best one regarding linearity and recoveries was 263.8 nm (Fig. 7a). For PHE, morl family with scale parameter of 50 was used on PHE/PHEdeg 30 ratio spectra, with several zero-crossing points obtained, the best wavelength was 282.6 nm (Fig. 7b).

### Comparative study

The two derivative methods (ND and CWT) showed the same performance regarding linearity and recoveries in laboratory prepared mixtures for PHE as shown in Table 1, while differences appeared between them in case of IBU. CWT showed better sensitivity regarding IBU and the linearity range extended from 20 to 180 µg/mL instead of 30–180 µg/mL in case of ND. This may be credited to the fact that CWT has amplification property that enhances the signal in contrast to ND which diminishes the signals, especially in higher orders as the fourth order in case of IBU. This fact was highlighted in previous studies on CWT [49, 50]. Another difference appears in mixture no. 12, as IBU recovery was very bad, which can be attributed to the small absorbance of IBU (30 µg/mL) in contrast to PHEdeg (25 µg/mL). This is due to the small absorbance of PHEdeg at 272.4 nm, which was not affecting recovery of IBU when present in high concentration compared to PHEdeg. This result shows the advantage of CWT over ND, as CWT gives variety of families and orders that make finding a true zero-crossing point possible in contrast to the simple algorithm of ND with only 4 derivatives orders to look for the zero-crossing points.

**Table 1:** Determination of IBU and PHE in laboratory prepared mixtures by the proposed methods

Mix. No	Concentration (µg/mL)			ND		CWT		DRZC		CWTR-ZC	
				Recovery %^b^							
	IBU	PHE	PHEdeg^a^	IBU	PHE	IBU	PHE	IBU	PHE	IBU	PHE
	120	3	0	99.23	98.22	98.45	98.30	NA	102.08	99.36	98.86
	60	4	0	100.51	102.30	98.36	98.78	NA	101.12	100.80	98.99
	90	30	0	100.43	99.33	97.94	99.26	NA	100.24	100.24	99.68
	150	7	0	100.51	101.90	99.85	100.87	NA	101.79	100.43	98.46
	180	20	0	101.58	101.30	99.31	100.60	NA	101.03	100.40	101.02
	120	3	1	100.00	101.07	100.20	98.59	NA	97.62	98.99	97.93
	120	3	3	99.23	101.43	99.16	99.17	NA	97.62	99.58	97.91
	60	40	10	102.95	100.25	101.05	102.61	NA	99.62	100.98	100.04
	20	20	10	NA	101.35	97.76	99.87	NA	101.47	98.64	100.65
	40	6	5	101.35	100.14	100.73	100.68	NA	98.66	100.36	98.80
	60	10	15	101.92	97.99	102.12	101.30	NA	100.71	101.60	101.56
	30	10	25	94.10^c^	98.84	101.58	98.36	NA	101.61	101.19	99.47
	150	10	25	100.36	98.31	100.35	102.27	NA	101.61	100.80	98.89
Mean ± SD				100.73 ± 1.13	100.19 ± 1.50	99.76 ± 1.40	100.05 ± 1.46	NA	100.40 ± 1.55	100.26 ± 0.88	99.40 ± 1.14

^a^Equivalent to PHE

^b^Average of three determinations

^c^Excluded according to the rejection rule [53]

Again, the two methods (DRZC and CWTR-ZC) showed the same performance regarding PHE in linearity and recoveries in laboratory prepared mixtures as shown in Table 1, while major differences appeared in case of IBU. DRZC method failed to find a suitable zero-crossing point for IBU, due to the limited options in its derivative calculation and the reduced wavelength range used (240–300 nm). On the contrast, CWT algorithm can use several wavelet families and orders to calculate the first order derivatives, which increase the possibility of finding zero-crossing points in limited range [51].

From the previous results, we can conclude that CWT technique represents a better option for solving the problem of minor components. It allows us to find the zero-crossing points between drugs in limited wavelength ranges, that will be obtained in several situations to solve the problem of minor components. This can be highlighted in the results for IBU, where ND failed to find zero-crossing points in ratio spectra data (DRZC method), while on raw data (ND method), 1st, 2nd and 3rd order derivatives failed to record a suitable zero-crossing point. In ND, we had to calculate the 4th order derivative to find this zero-crossing point. This has diminished the signal and decreased the sensitivity of the method compared to CWT, which allowed a zero-crossing point directly and having the property of amplification, preserved the signal and increased the sensitivity of the method.

### Validation and application to pharmaceutical formulation

The methods were validated according to ICH guideline Q2(R1) [52]. Accuracy was assessed using three concentrations of each drug (80, 110, 140 μg/mL for IBU and 15, 25, 35 μg/mL for PHE) with three replicates for each concentration and mean recoveries were calculated which ranged from 100.20% to 100.59% showing acceptable accuracy (Table 2). Repeatability and intermediate precision were measured three times on the same day and on different three days, respectively, using three concentrations of each drug (90, 120, 150 μg/mL for IBU and 10, 20, 30 μg/mL for PHE). RSD of the responses were calculated and did not exceed 2% as shown in Table 2, proving that the method was precise.

**Table 2:** Validation sheet of the proposed methods for the simultaneous determination of the binary mixture

Parameter	ND		CWT		DRZC	CWTR-ZC	
	IBU	PHE	IBU	PHE	PHE	IBU	PHE
Accuracy^a^	100.53 ± 0.97	100.31 ± 1.10	100.32 ± 0.86	100.59 ± 0.60	100.20 ± 1.06	100.54 ± 1.10	100.33 ± 1.22
Specificity^b^	100.73 ± 1.13	100.19 ± 1.50	99.76 ± 1.40	100.05 ± 1.46	100.40 ± 1.55	100.26 ± 0.88	99.40 ± 1.14
Precision							
Repeatability^c^	0.675	0.774	0.419	0.682	0.882	0.443	0.824
Intermediate precision^d^	1.027	1.264	0.924	1.086	1.339	1.030	1.239
Robustness^e^ (± 0.2 nm)	1.972	0.394	1.690	1.208	0.512	1.879	1.665
Linearity							
Slope	0.0130	− 0.0934	0.0007	0.0431	0.0224	0.0014	0.0184
Intercept	− 0.0070	− 0.0088	0.0003	− 0.0075	0.0034	− 0.0020	0.0042
Correlation coefficient (r)	0.9999	0.9998	0.9999	0.9999	0.9998	0.9996	0.9995
Range (μg/mL)	30–180	3–40	20–180	3–40	3–40	20–180	3–40

^a^The accuracy (n = 3), mean recovery of three concentrations (80, 110, 140 μg/mL) for IBU and (15, 25, 35 μg/mL) for PHE

^b^Specificity is the mean R% ± SD of the two drugs in laboratory prepared mixtures

^c^The intraday (n = 3), RSD of three concentrations (90, 120, 150 μg/mL) for IBU and (10, 20, 30 μg/mL) for PHE repeated three times within day

^d^The interday (n = 3), RSD of three concentrations (90, 120, 150 μg/mL) for IBU and (10, 20, 30 μg/mL) for PHE repeated three times in three days

^e^Robustness (*n* = 3), RSD of the recovery calculated at different wavelengths for three concentrations (90, 120, 150 μg/mL) for IBU and (10, 20, 30 μg/mL) for PHE

The linearity of the methods was assessed using at least 6 points calibration for each method and correlation coefficients ranging from 0.9995 to 0.9999 proved the linearity of the proposed methods. The linearity was in the range 3–40 μg/mL for PHE in all methods, while for IBU it was 20–180 and 30–180 μg/mL in CWT and ND methods, respectively. Specificity of the methods was evaluated by calculating the mean R% ± SD of the drugs in their laboratory prepared mixtures to measure their ability to quantitate the analytes with sufficient discrimination. The results in Table 2 show the methods were specific for each drug in presence of the other drug and PHEdeg.

The robustness of the methods was measured using the new approach introduced in previous work [50], where the recoveries were calculated at ± 0.2 nm from the zero-crossing points. From robustness results, we can observe better RSD% in case of wavelengths in broad horizontal bands on the spectrum as PHE in ND and DRZC methods (Figs. 4a and 6), in contrast to a steep portion of the spectrum as IBU in ND and CWTR-ZC (Fig. 4b and Fig. 7a).

The CWT algorithm showed better performance in derivative and derivative ratio methods, so it was used successfully for determination of the two drugs in Grippostad® tablets as shown in Table 3. The results of analysis of IBU and PHE by pharmacopeial methods in pure powder were statistically compared with CWT and CWTR-ZC methods and showed no significant difference as shown in Table 4.

**Table 3:** Determination of IBU and PHE in Grippostad^®^ tablets by the proposed CWT methods

Drug	CWT	CWTR-ZC
	Recovery % ± SD^a^	
IBU (120 µg/mL)	101.13 ± 1.24	100.32 ± 0.79
PHE (3 µg/mL)	98.94 ± 0.99	99.72 ± 0.93

^a^Average of three determinations

**Table 4:** Statistical comparison for the results obtained by the proposed CWT methods and the pharmacopeial methods [24] for the analysis of IBU and PHE in pure powder

Parameter of interest	CWT		CWTR-ZC		Pharmacopeial methods	
	IBU	PHE	IBU	PHE	IBU^a^	PHE^b^
Mean	99.26	99.56	99.61	99.4	99.58	100.02
SD	0.93	1.13	0.81	1.01	1.10	0.86
N	5	5	5	5	5	5
Variance	0.874	1.271	0.656	1.012	1.199	0.737
Student’s t test^c^ (2.306)	0.500	0.723	0.049	1.045	–	–
F value^c^ (6.39)	1.373	1.724	1.829	1.372	–	–

^a^Acid–base titration with sodium hydroxide standard and phenolphthalein indicator

^b^Potentiometric titration with ethanolic sodium hydroxide standard

^c^The values in the parenthesis are the corresponding theoretical values of t and F at P = 0.05

## Conclusion

Four signal processing methods (ND, CWT, DRZC and CWTR-ZC) were developed and compared in the analysis of the ternary mixture of IBU, PHE and PHEdeg. The comparative study performed suggested that methods based on CWT algorithm show better alternative to ND in quantitation of pharmaceutical mixtures with minor components. They allow higher flexibility in calculating the transformed spectra giving more opportunities to find zero-crossing points in limited wavelength range usually associated with minor components. The CWT methods were successfully applied for the analysis of the two drugs in their pharmaceutical formulation, suggesting the suitability and validity for application of these methods in quality control laboratories to solve the problem of minor components in spectrophotometric analysis.

## Data Availability

The data analyzed during the current study are available from the corresponding author on reasonable request.

## Abbreviations

CWT: Continuous wavelet transform
CWTR-ZC: Continuous wavelet transform ratio-zero crossing
DRZC: Derivative ratio-zero crossing
DS: Derivative spectrophotometry
gaus-5: Gaussian-5 wavelet family
IBU: Ibuprofen
ICH: International Council for Harmonisation
morl: Morlet wavelet family
ND: Numerical differentiation
PHE: Phenylephrine
PHEdeg: PHE degradation products
